# Valorization of Blackcurrant Pomace for the Development of Functional Stirred Yogurt with Enhanced Nutritional and Antioxidant Properties

**DOI:** 10.3390/foods14213650

**Published:** 2025-10-26

**Authors:** Florin Daniel Lipșa, Roxana Nicoleta Rațu, Florina Stoica, Iuliana Motrescu, Irina Gabriela Cara, Ramona-Maria Cristea, Eugen Ulea

**Affiliations:** 1 Department of Food Technologies, Faculty of Agriculture, “Ion Ionescu de la Brad” Iasi University of Life Sciences, 3 Sadoveanu Alley, 700490 Iasi, Romania; florin.lipsa@iuls.ro; 2 Department of Pedotechnics, Faculty of Agriculture, “Ion Ionescu de la Brad” Iasi University of Life Sciences, 3 Sadoveanu Alley, 700490 Iasi, Romania; florina.stoica@iuls.ro; 3 Department of Exact Sciences, Faculty of Horticulture, “Ion Ionescu de la Brad” Iasi University of Life Sciences, 3 Sadoveanu Alley, 700490 Iasi, Romania; 4 Research Institute for Agriculture and Environment, “Ion Ionescu de la Brad” Iasi University of Life Sciences, 3 Sadoveanu Alley, 700490 Iasi, Romania; irina.cara@iuls.ro; 5 Department of Agricultural Science and Food Engineering, “Lucian Blaga” University of Sibiu, 7-9 Dr. Ion Ratiu, 550024 Sibiu, Romania; 6 Department of Plant Science, Faculty of Agriculture, “Ion Ionescu de la Brad” Iasi University of Life Sciences, 3 Sadoveanu Alley, 700490 Iasi, Romania; eugen.ulea@iuls.ro

**Keywords:** waste valorization, antioxidants, sustainable food processing, dairy products

## Abstract

In light of the growing concerns of consumers who are increasingly turning towards healthier food options, both researchers and producers in the food industry are exploring the use of agro-industrial by-products as nutritionally valuable ingredients. This strategy not only enables the development of value-added food products, but also supports sustainability through the valorization of waste. Blackcurrant pomace (BP), a by-product obtained after juice extraction, has been shown to be rich in bioactive compounds, dietary fiber, antioxidants, and anthocyanin pigments. For these reasons, the innovative aspect of the study was its use of different proportions of BP powder, 5%, 10%, and 15%, when obtaining new varieties of stirred yogurt. This study assesses the impact of BP powder on the stirred yogurt’s antioxidant content, physicochemical properties, color, microbiological characteristics, and sensory qualities. The findings showed that BP powder intensified the yogurts’ coloration and considerably improved their antioxidant activity (which ranged from 8.21 ± 0.35 to 21.15 ± 0.49 µmol TE/g DM) and nutritional quality. The panelists’ positive acceptance was confirmed by sensory evaluation, and the 10% BP formulation (DBBP2) was rated as the most favorable. These results show that BP is a valuable ingredient for enhancing dairy products, creating nutritious, appealing yogurts while promoting sustainable food production and valorization of food waste.

## 1. Introduction

The global food industry produces millions of tons of by-products and waste every year. These by-products are challenging to dispose of but offer significant opportunities for sustainable valorization. They are often rich in valuable components such as proteins, dietary fibers, antioxidants, and natural sugars. As a result, both industry and academia are becoming more interested in finding ways to valorize these valuable by-products, especially as sources of bioactive compounds that can be used in food, drugs, and cosmetics [1,2,3].

Blackcurrant (*Ribes nigrum* L.), a deciduous shrub from the *Grossulariaceae* family, is native to northern and central Europe and northern Asia. It is currently the second most cultivated berry crop in Europe [4]. Despite it being an abundant source of bioactive phytochemicals, including phenolic acids, anthocyanins, flavonols, hydrolysable and condensed tannins, fresh blackcurrant consumption is limited due to its intensely astringent taste, sour flavor, and short shelf life [5,6,7].

During juice processing, a considerable amount of blackcurrant pomace (BP), the fibrous residue consisting of skins, seeds, and pulp, remains as a by-product. Owing to its high amounts of dietary fiber, organic acids, and anthocyanins, BP has gained increasing attention as a functional ingredient with promising applications in the formulation of health-promoting food products [8,9,10].

Among various underutilized berry crops, such as elderberry and chokeberry, blackcurrant stands out as an especially rich source of natural antioxidants, including anthocyanins and vitamin C. While other berries also contain significant levels of bioactive compounds, blackcurrant is particularly noteworthy for its high antioxidant capacity and potential functional applications [11]. In countries such as Romania, it is predominantly cultivated by local producers for direct consumption or processing into jams, jellies, syrups, and juices, though its broader economic significance remains limited [12].

In the context of rising environmental concerns and the urgent need to reduce food waste, the valorization of agro-industrial by-products such as BP has become increasingly important. BP, often discarded during juice production, is now recognized for its wealth of bioactive compounds, most notably polyphenols, anthocyanins, flavonoids, and dietary fiber, which are associated with numerous health benefits, including antioxidant, anti-inflammatory, and antimicrobial properties [13,14]. As a result, BP is being investigated as a promising functional ingredient that could be incorporated into foods and beverages.

Scientific progress has shown that fruit pomace can be effectively incorporated into a variety of value-added food products. Applications include its use as natural colorants [15], antioxidant sources and pectins [16], as well as its use in the formulation of innovative food products such as jelly desserts [17], whey-based beverages [18], yogurts [19], and smoothies [20].

Yogurt is a product recognized for both its nutritional value and its multiple beneficial effects on health, making it one of the most consumed fermented dairy products worldwide [21]. Numerous studies have demonstrated that regular consumption of yogurt containing live cultures and probiotic strains can help lower blood cholesterol levels and improve tolerance in individuals with lactose intolerance [22]. Farvin et al. [23] demonstrated that the antioxidant activity of yogurt is associated with antioxidant peptides generated by lactic acid bacteria during the fermentation process. It has also been shown that yogurt can improve various conditions, such as intestinal infections, inflammation, diarrhea, and even colon cancer [24].

Additionally, recent research has demonstrated that the health benefits of yogurt can be amplified by adding dietary fiber and sources of bioactive compounds or antioxidants [25]. Supplementing yogurt with fruits improves its functional properties by increasing antioxidant activity and enhancing sensory characteristics, giving it a more pleasant taste and a more attractive texture. The antioxidants present in fruits, carotenoids, flavonoids, anthocyanins, and other polyphenols play an important role in protecting cells against oxidative stress, thus contributing to reducing chronic disease incidence [26].

Our study aimed to investigate the incorporation of blackcurrant pomace (BP) powder into stirred yogurt at varying concentrations, with a focus on evaluating its influence on a wide range of quality parameters. Specifically, the study assessed mineral composition, color stability, microstructure, microbial viability, antioxidant potential, physicochemical and phytochemical characteristics, and sensory attributes. Additionally, our research explored the stability of bioactive compounds during both processing and storage. By examining these factors, this study provides valuable insights into the functional and technological potential of BP as a sustainable ingredient in the dairy industry, promoting the effective valorization of agro-industrial by-products and contributing to a circular food economy.

## 2. Materials and Methods

### 2.1. Reagents and Chemicals

The following chemicals were sourced from Sigma Aldrich Steinheim (Darmstadt, Germany): Folin–Ciocalteu reagent, 1% citric acid, 70% ethanol, 6-hydroxy-2,5,7,8-tetramethylchromane-2-carboxylic acid (Trolox), gallic acid, methanol, 2,2-diphenyl-1-picrylhydrazyl (DPPH), sodium hydroxide, sodium nitrite, potassium chloride solution, sodium acetate solution, aluminum chloride, and sodium carbonate. 

### 2.2. BP Powder Preparation

Blackcurrants (*Ribes nigrum* L.) were acquired from a supermarket in Iasi, Romania, in June 2024. Fresh pomaces consisting of peels, residual pulp, and seeds were obtained directly after juice pressing of blackcurrants from Bosch MES3500, a commercial juice manufacturer run by Philips Consumer Lifestyle B.V., Drachten, Holland.

The blackcurrant pomace (BP) was then stored at −25 °C for freezing. Following this, frozen BP underwent freeze-drying using a BIOBASE BK-FD10T (Jinan, China) at a temperature of −42 °C and a pressure of 0.10 mBar for 50 h.

After drying, the pomace was ground into a fine powder (sieved using a 0.2 mm screen) using a household electric grinder (MC 12, Producer Stephan, Hameln, Germany) and stored in polyethylene bags. Prior to being incorporated into the yogurt, the BP powder was decontaminated through a UV laser treatment. The BP powder was exposed to UV-C for 3–4 min at a dose of 25–27 J/mL, with a lamp intensity of approximately 10 mW/cm^2^. At these levels, the impact on bioactive compounds was minimal, as UV treatment is a non-thermal process that preserves the functional integrity of most phytochemicals.

### 2.3. Extraction of Phytochemicals from BP Powder

Extraction of bioactive compounds from BP powder was performed using ultrasound-assisted extraction, a method outlined by Kruszewski et al. [9], with minor adjustments. Firstly, 1 g of BP powder was mixed with 9 mL of 70% ethanol acidified with 1 mL of 1% citric acid solution. The mixture was subjected to ultrasound treatment using an ultrasonic bath (Elmasonic S 180 H, Elma, Singen, Germany) for 55 min at 40 kHz, with a power of 100 W at 30 ± 5 °C.

Afterward, the mixture was centrifuged for 5 min at 6500 rpm and 4 °C. The supernatant was transferred to a separate vial and kept in the dark at 4 °C for subsequent analyses.

### 2.4. BP Extract Characterization

The powdered blackcurrant extract was characterized in terms of anthocyanin, flavonoid, and polyphenol content and antioxidant activity.

#### 2.4.1. Total Anthocyanins Content

The total anthocyanin content was measured spectrophotometrically using the pH differential method established by Giusti and Wrolstad [27]. Thus, 200 µL of each sample was transferred to cuvettes, followed by the addition of 800 µL of buffer solution at pH 1.0 (KCl). Likewise, 800 µL of buffer solution at pH 4.5 was added to separate cuvettes.

The absorbance was measured independently and in triplicate at wavelengths of 510 nm and 700 nm using a spectrophotometer (Analytik Jena Specord 210 Plus, Jena, Germany), after the samples had been kept in the dark for 15 min at room temperature.

The findings were expressed as milligrams of cyanidin-3-glucoside (C3G) per gram of dry matter (DM). The total anthocyanin content (C) was calculated using the following Formula (1):

(1) $$C\;(mg\;C3G/g\;DM)=\frac{A\times MW\times DF}{\varepsilon \times l\times m}$$

where A = (A520 nm − A700 nm) pH 1.0 − (A520 nm − A700 nm) pH 4.5. The molecular weight (Mw) of cyanidin-3-glucoside is 449.2 g/mol, and the path length (l) is 1 cm. The dilution factor (Df) and the amount of sample (m) are taken into account during the analysis. The molar extinction coefficient (ε) for cyanidin-3-glucoside is 26,900 L/mol/cm.

#### 2.4.2. Total Flavonoids Content

The AlCl_3_-based colorimetric method described by Rusu et al. [6] was used, with modifications, to determine the total flavonoid content of the BP extract.

In brief, a mixture consisting of 500 µL of BP extract, 2000 µL of ultrapure water and 150 µL of 5% NaNO2 solution was allowed to react for 6 min.

Then, 150 µL of 10% aluminum chloride solution was added to the mixture. After another 6 min, 1000 µL of 1 M NaOH solution was added.

The absorbance of the mixture was measured immediately at 510 nm. The total flavonoid content was determined by plotting a quercetin calibration curve, and the results were expressed as milligrams of quercetin equivalents (QEs) per gram of DM.

#### 2.4.3. Total Polyphenols Content

To determine the total polyphenol content of the BP extract, the Folin–Ciocâlteu colorimetric method was applied, following the procedure outlined by Stoica et al. [19]. Briefly, 200 μL of extract, 15.8 mL of distilled water, and 1 mL of Folin–Ciocâlteu reagent were mixed. After 10 min, 3 mL of a 20% sodium carbonate solution was added, and the mixture was kept in the dark at room temperature for 60 min. The absorbance was then measured at 765 nm using a spectrophotometer (Analytik Jena Specord 210 Plus, Jena, Germany). A standard curve was prepared using different concentrations of gallic acid, and the results were expressed as mg gallic acid equivalents (GAEs) per g DM.

#### 2.4.4. DPPH Scavenging Activity Method

The 2,2-diphenyl-1-picrylhydrazyl (DPPH) antioxidant activity method of the BP extract was evaluated based on its ability to scavenge the DPPH radical, following the method described by Gavril et al. [28]. Briefly, 3.90 mL of methanolic DPPH solution (0.1 M) was mixed with 0.10 mL of the BP extract, and the mixture was allowed to react for 30 min at 25 °C in the dark. For the blank sample, 0.10 mL of methanol was combined with 3.90 mL of 0.1 M DPPH solution. The absorbance was measured at 515 nm using a UV–visible spectrophotometer (Analytik Jena Specord 210 Plus, Jena, Germany). A standard curve was generated using various concentrations of Trolox. The antioxidant activity was expressed as µmol of Trolox equivalent (TE) per gram of DM. Additionally, the radical scavenging activity was calculated as the percentage of inhibition using Equation (2):

(2) $$\mathrm{DPPH}\;\mathrm{scavenging}\;\mathrm{activity}\;(\%)\;=\;\frac{A\;control-A\;sample}{A\;control}\times 100$$

where A sample is the absorbance value of the DPPH solution mixed with BP extract; A control is the absorbance value of the DPPH solution only.

### 2.5. Preparation and Characterization of BP-Supplemented Stirred Yogurt

The raw milk (500 L cow’s milk, provided by Rediu farm) was standardized to a fat content of 3% and concentrated by heating at 90 °C for 30 min to increase the total solids content and improve the final consistency of the final product. The milk was subsequently cooled to 44 ± 0.05 °C in a double-jacketed vat with temperature control to ensure uniform heat transfer and minimize thermal stress on the starter culture (*Streptococcus thermophilus* and *Lactobacillus delbruckii* subsp. *bulgaricus*, YF-L812 commercial product, Chr. HANSEN, Hørsholm, Denmark). It was then inoculated with 3% (*v*/*v*) yogurt starter culture and incubated at 42 ± 0.05 °C until the pH reached 4.60, corresponding to gel formation.

Fermentation was terminated by gradually cooling the product to 20 ± 0.5 °C, followed by further cooling to 4 ± 0.5 °C to stabilize the gel matrix and suppress microbial activity. The coagulated mass was gently stirred with a sterilized stainless-steel paddle to obtain the homogeneous texture characteristic of stirred yogurt. Experimental batches were then prepared by adding the respective powders and homogenizing, as detailed in Table 1.

All formulations were aseptically filled into 200 mL pre-sterilized glass containers with airtight closures and stored at 4 ± 0.01 °C for 21 days. The day following manufacture was considered day 1 of storage. Analyses were performed in triplicate on days 1 and 21 to evaluate product quality and stability.

### 2.6. Characterization of Physicochemical and Phytochemical Properties of BP-Stirred Yogurt

The physicochemical properties of the control and supplemented stirred yogurt samples, including moisture, dry matter, fat, proteins, fiber, and ash, carbohydrates, and energy values, were assessed following standardized AOAC procedures [29].

Ash content was measured using a Caloris CL 1206 oven (Romania) following ISO 2171:2009 [30]. Dry matter was assessed using the drying oven method at 103 °C (Memmert ULM500, Uden, The Netherlands). Fat content was measured by extraction with organic solvents following SR EN ISO 6492:2010 [31] using a Soxhlet automatic extraction system (SER 148/3, Velp Scientific, Usmate, Italy). Protein content was measured using the Kjeldahl method in accordance with SR EN ISO 5983–2:2009 [32], with an automated nitrogen analyzer (UDK 149, Velp Scientific, Milan, Italy). Crude fiber was assessed by acid and alkali digestion according to ISO 6865:2001 [33] using an automatic analyzer (Fibertec 2010, Tecator, Höganäs, Sweden).

(3) $$\mathrm{Energy}\;\mathrm{value}=\left(\%\;crude\;protein\times 4\right)+\left(\%\;crude\;fat\times 9\right)+\left(\%\;carbohydrates\times 4\right)+(\%\;\mathrm{crude}\;\mathrm{fiber}\;\times \;2)$$

According to the AOAC [29] protocols, pH was determined using a pH meter (WTW InoLab, Xylem Analytics GmbH, Weilheim, Germany), and total titratable acidity (TTA) was determined by the titrimetric method. The results were expressed as a percentage of lactic acid. Total soluble solids (TSSs) were measured using a PR-101 digital refractometer. The results were expressed in ºBrix.

The total anthocyanins, flavonoids, polyphenolic content, and antioxidant activity of the stirred yogurt enriched with BP powder were determined using the methods outlined in Section 2.4.

### 2.7. Color Evaluation of Supplemented Stirred Yogurt

Color analysis of the control and supplemented stirred yogurts was conducted using a Konica Minolta CR1 10 colorimeter (Konica Minolta Solutions Ltd., Basildon, UK). The color parameters L* (lightness/darkness; 0 to 100), a* (red/greenness), and b* (yellow/blueness) of the samples were assessed using the CIE Lab* color system, as defined by the International Commission on Illumination (CIE).

The primary data for Hue Angle (visual color appearance), ΔE (total color difference), and Chroma (color intensity) were calculated using the formulas provided below.

Hue angle = arctan (b*/a*) for quadrant I (+a*, +b*).

Hue angle = 360 + arctan (b*/a*) for quadrant IV (+a*, −b*).

Chroma = $\sqrt{{{(a}^{*})}^{2}+{{(b}^{*})}^{2}}$.

ΔE = ($\sqrt{{{(L}^{*}-L_{0})}^{2}+{{(a}^{*}-a_{0})}^{2}+{{(b}^{*}-b_{0})}^{2}}$), L_0_, a_0_, b_0_ = blank value of each sample [34].

### 2.8. Mineral Evaluation of Supplemented Stirred Yogurt

The mineral content of the BP powder and obtained stirred yogurts was determined using atomic absorption spectrometry (ContrAA 700, Analytik Jena, Jena, Germany) with a flame atomizer system [25]. Mineral contents (Ca, P, K, Mg, Zn, Fe, Cu, and Na) in both BP powder and stirred yogurt samples were assessed using a MiniWAVE Microwave digestion system (SCP Science, Baie D’Urfé, QC, Canada) equipped with a 50 mL Teflon vessel. A 1 g sample was placed in the Teflon vessel and digested with 10 mL of nitric and hydrochloric acids (8:2) at a temperature of 180 °C, a digestion time of 20 min, and a microwave power of 1000 W. After digestion, the sample was cooled, transferred into a 50 mL volumetric flask, and diluted with ultrapure water to the mark. A blank sample was included in each digestion run. Each sample was tested in triplicate, and the results were expressed as mg per 100 g of dry matter (DM).

### 2.9. Scanning Electron Microscopy Analysis

The morphology of the BP powder and obtained stirred yogurts and their elemental composition were characterized with a scanning electron microscope (SEM) (Quanta 450, FEI, Thermo Fisher Scientific, Hillsboro, OR, USA) and an energy-dispersive X-ray detector (EDS) (EDAX, AMETEK Inc., Berwyn, PA, USA). Prior to analysis, calibration was carried out using a standard AlCu sample, which consisted of copper foil mounted on an aluminum grid. The EDS spectra analysis was performed using the TEAM version V4.1 system from EDAX Inc. (Mahwah, NJ, USA). Each of the samples were analyzed under low-vacuum circumstances, with a pressure of roughly 6.1 × 10^−4^ Pa, an electron acceleration voltage of 15 kV, and a 500× (50 μm) magnification.

### 2.10. Microbiological Analyses

The microbiological analysis of the samples was conducted under sterile conditions immediately post-processing (day 0) and after 21 days of storage at 4 °C. For each analysis, 10 mL of the stirred yogurt was homogenized with 90 mL of buffered peptone water using a laboratory blender (Seward, Worthing, UK) at 250 rpm for 5 min. Subsequent serial dilutions were conducted by transferring 1 mL from the preceding dilution into 9 mL of buffered peptone water. Bacteria, yeasts, and molds were identified from these dilutions utilizing standard spread and pour plate methodologies [35].

The microbiological media utilized for inoculation comprised non-selective Plate Count Agar (PCA; Scharlau, Barcelona, Spain), Potato Dextrose Agar (PDA; Scharlau, Barcelona, Spain), and selective chromogenic agars, including Rapid E. coli 2 (RE; Bio-Rad Laboratories, Marnes-la-Coquette, France) and Rapid Staph (RS; Bio-Rad, Marnes-la-Coquette, France). The total count of aerobic mesophilic bacteria was determined on PCA plates during a 72 h incubation at 30 °C. Yeasts and molds were enumerated on PDA plates after a 5-day incubation at 28 °C. *Escherichia coli* and other coliforms were evaluated using RE medium after 24 h of incubation at 37 °C. Furthermore, all samples were cultured on RS medium to identify and quantify coagulase-positive staphylococci (*Staphylococcus aureus*) and incubated for 24 h at 37 °C. Following incubation, colony counts were conducted utilizing an automatic colony counter (Scan 1200, Interscience, Saint-Nom-la-Bretèche, France), with findings presented as logarithmic values of colony-forming units per gram (log CFU g^−1^) [36].

### 2.11. Sensory Evaluation of Supplemented Stirred Yogurt Samples

The sensory evaluation of the stirred yogurt samples was realized by 20 panelists from the Faculty of Agriculture at the University of Life Sciences (Iasi, Romania). These panelists, all experts in food technology, were selected based on their willingness to participate and their knowledge of dairy products. They were regular consumers of stirred yogurt and had no allergies to it. Prior to the evaluation, the panelists underwent two training sessions to familiarize themselves with the sensory descriptors, including changes in color, texture, and flavor of the stirred yogurt samples. The assessment included appearance, color, flavor, texture, taste, odor, aftertaste, and overall acceptability, rated on a nine-point scale (1—extreme dislike; 9—extreme like) [37]. The samples were presented in clear, colorless plastic containers marked with a three-digit code. To cleanse their palates between samples, panelists were provided with water and bread. They were also encouraged to note any observations or feedback on the obtained products.

### 2.12. Statistical Analysis

The results were presented in triplicate as the mean ± standard deviation (SD) for each sample. Statistical differences among the all samples were analyzed using Minitab 19.0 (free trial). When significant differences were observed, one-way analysis of variance (ANOVA) followed by Tukey’s test with a 95% confidence interval was applied. Principal component analysis (PCA) was performed on the descriptive sensory data using XLSTAT, an add-in software for Microsoft Office Excel (Trial Version 2024, Addinsoft, Paris, France).

## 3. Results and Discussion

### 3.1. Phytochemical Characterization of BP Powder

Since most phenolic compounds are primarily located in the skins, berry pomaces retain a variety of phenolic compounds with significant antioxidant potential, particularly high concentrations of anthocyanins. These compounds exhibit strong antimicrobial, anti-mutagenic, and anti-inflammatory properties. Extracting these bioactive compounds from berry pomaces provides a source of natural colorants and active ingredients for use in the food and pharmaceutical industries [38].

Table 2 presents the phytochemical content (polyphenols, flavonoids, anthocyanins and antioxidant activity), physicochemical, mineral and color properties of BP powder.

Sójka and Król [39] reported that the anthocyanin content in BP ranged from 3.44 to 10.46 mg/g, depending on the year of the fruit harvest. Additionally, Gagneten et al. [8] found 18.0 mg of cyanidin-3-glucoside per gram of dry weight in the dehydrated blackcurrant by-product from juice production. Blejan et al. [40] reported a Trolox equivalent DPPH radical scavenging capacity of 25.5 ± 0.88 μmol/g dry extract of BP.

Basegmez et al. [41] reported a total phenolic content of 24.34 mg GAE/g in BP, whereas Gagneten et al. [8] found a higher content of 37.5 mg GAE/g dry weight in the blackcurrant by-product.

The BP exhibited a dark red/purple color, reflecting its high anthocyanin content. The powder was situated in quadrant I (+a*, +b*), as indicated by the color indices.

The chemical composition of BP highlights its notable nutritional value. Its relatively low moisture content supports good storage stability, while its ash content reflects a valuable mineral profile. The presence of lipids contributes to its energy value and potential functional properties, and its protein content indicates its role as a modest source of plant-based proteins. Moreover, BP is particularly rich in dietary fiber, emphasizing its potential for use in functional food formulations aimed at promoting digestive health [12,40]. The chemical composition of BP in a study by Sankowski et al. [42] revealed protein content of 17.4 ± 0.2%, 16.2 ± 0.1% fat, dry matter content of 94.9 ± 0.5%, and 58.6 ± 0.2 g/100 g insoluble dietary fiber.

The mineral analysis of BP powder indicates that blackcurrant by-products may serve as a valuable source of calcium, potassium, magnesium, potassium, iron, and phosphorus [42,43,44].

The composition of blackcurrant by-products is influenced by the variety, regional agronomic circumstances, extraction methods (including solvent type, temperature, pH, and light intensity), and assessment procedures employed [9,41,45]. The extraction of bioactive compounds from dried pomace samples of wild blackcurrants and blackberries was carried out using methanol as the solvent in an ultrasonic bath for 60 min at room temperature. The antioxidant capacity, evaluated through DPPH radical scavenging activity, revealed the highest values for bilberry pomace bilberries (26.1 ± 1.07 μmol TE/g DM), followed by blackcurrant (25.5 ± 0.88 μmol TE/g DM) and blackberry (19.2 ± 0.65 μmol TE/g DM) [40].

Research indicates that BP extracts serve as a superior source of phytochemicals with antioxidant properties and can be used in food products to reduce agro-industrial residues.

### 3.2. Characterization of Bioactive Potential of Supplemented Stirred Yogurts and Storage Stability of the Samples

BP is a valuable by-product of the food industry, which is high in bioactive compounds, especially anthocyanins, and can be used further, such as in pigment extraction or food coloring [46]. The phytochemical characteristics and DPPH radical scavenging ability of control and experimental stirred yogurt are reported in Table 3.

The incorporation of BP powder into the stirred yogurt samples led to elevated amounts of anthocyanins, flavonoids, and polyphenols. Furthermore, the experimental samples exhibited significant bioactive content and displayed remarkable antioxidant activity levels. On day 0, significant statistical differences (*p* < 0.05) were observed among all stirred yogurt samples (DBC, DBBP1, DBBP2, and DBBP3) across all measured phytochemical characteristics and antioxidant activity. For total anthocyanins, DBBP3 exhibited the highest content (98.44 ± 0.41 mg C3G/100 g DM), while DBBP1 had the lowest (33.42 ± 0.26 mg C3G/100 g DM), with all samples differing significantly. In antioxidant activity, DBBP3 recorded the highest value (21.15 ± 0.49 µmol TE/g DM), while DBC had the lowest (8.21 ± 0.35 µmol TE/g DM), again with all samples showing statistically significant differences. These results confirm that increased BP supplementation significantly enhanced the bioactive potential of the enhanced dairy stirred yogurts. Similar behavior has been reported for other fruit pomaces incorporated in fermented dairy matrices. Apple pomace powders and syrups have repeatedly enhanced yogurt antioxidant capacity and consumer acceptance, while also improving texture/viscosity owing to fiber-driven water binding [21,47,48]. Smoothies with 6% dried BP contained a lower anthocyanin content of 9.65 ± 0.10 mg/100 mL and higher total polyphenolic compounds of 158.72 ± 3.58 mg/100 mL [18].

Bertolino et al. [49] investigated the impact of incorporating hazelnut powder directly into yogurt. Their findings showed a substantial enhancement in both radical scavenging activity and total phenolic content, with average increases of 96% and 31%, respectively, compared to the control sample. Extracts derived from pomegranate skin and mesocarp have been reported to enhance the antioxidant activity of buffalo and cow cheese formulations [50]. Similarly, Manzoor et al. [51] proposed incorporating papaya peel powder into yogurt formulations to create a functional product with lower syneresis, enhanced nutritional quality, and improved antioxidant properties. Also, total phenol content (50 µg GAE /mL) and total anthocyanin content (15 mg C3G/mL) in yogurt beverage containing BP were higher than the plain yogurt (control) [17]. Collectively, these studies situate BP alongside other pomaces as an efficacious fortifier of antioxidant potential and textural attributes, with BP’s high anthocyanin and fiber load offering a performance that is at least comparable and, in strongly pigmented products, potentially superior on a per-mass basis.

The dairy stirred yogurts’ phytochemical contents and antioxidant activity decreased over time (*p* < 0.05) but remained higher than the control. For total anthocyanins, DBBP3 still retained the highest content after 21 days (94.21 ± 0.33 mg C3G/100 g DM), while DBBP1 had the lowest (29.39 ± 0.20 mg C3G/100 g DM). The decrease in anthocyanins over time was significant in all supplemented samples. In terms of total flavonoids, DBBP3 remained the highest (102.82 ± 1.28 mg CE/100 g DM at day 21), while DBC had the lowest (51.04 ± 1.10 mg CE/100 g DM). Similarly, total polyphenols decreased slightly but significantly over time, with DBBP3 still having the highest content (167.54 ± 1.22 mg GAE/100 g DM) and DBC the lowest (84.61 ± 1.06 mg GAE/100 g DM) after storage. For antioxidant activity, all formulations experienced a decline, but DBBP3 maintained the highest value at day 21 (18.07 ± 0.27 µmol TE/g DM), whereas DBC dropped to the lowest (5.11 ± 0.20 µmol TE/g DM).

This loss can be ascribed to the reduced concentration of polyphenolic chemicals in the products; this statement is also supported by Szydłowska et al. [18]’s study. These consistent trends indicate that while storage negatively affected all bioactive compounds, higher levels of BP supplementation (especially 15%) significantly improved stability and preserved functional properties over the 21-day period.

Montibeller et al. [52] discovered that anthocyanin fortification from grape skin served as a natural pigment in the dairy product kefir. This study found that the enriched kefir preserved more than 50% of total anthocyanins for the initial 27 days, exhibiting around 67% retention on day 16.

For berry-fortified fermented milks, several reports document color/anthocyanin degradation kinetics extending to 28 days, typically first-order and influenced by pH, starter culture, oxygen, and light: anthocyanin content and a* values usually decrease significantly in the first two weeks and continue to decline thereafter [53,54,55]. In kefir/yogurt systems, anthocyanin decay half-lives of ~3–4 weeks have been reported, implying that, without additional stabilization (e.g., oxygen/light control, chelators, co-pigmentation, or encapsulation), functional color and antioxidant metrics will likely continue to diminish beyond 21 days [52,56]. Conversely, some matrices show gradual rises in global antioxidant measures (e.g., ABTS/DPPH) during storage due to proteolysis and peptide release, even as anthocyanins decline, underscoring the need to track multiple assays [57]. Future work could therefore extend monitoring to ≥28–35 days and model kinetics (k, t^1/2^) under varied packaging, headspace O_2_, and light exposure to define label-relevant shelf-life limits for both color and antioxidant functionality.

The concentration of anthocyanins diminished progressively during storage to various levels. Additionally, the decrease in antioxidant activity resulting from protein–polyphenol interaction is accomplished by diminishing the quantity of free hydroxyl radicals [58]. Raikos et al. [17] reported that polyphenols, including anthocyanins and tannins, interact with milk proteins, particularly caseins, through hydrophobic and hydrogen bonding. This interaction leads to the formation of non-covalent complexes that may reduce the measurable free polyphenol content but can also protect these compounds from early degradation during digestion, thereby potentially altering their release and absorption kinetics. In addition, BP represents a source of both condensed and hydrolysable tannins, which can form insoluble complexes with proteins and chelate divalent cations such as iron, zinc, and calcium, potentially influencing mineral bioavailability [40,59]. All these interactions may have both beneficial and adverse nutritional implications depending on the digestive environment, and their quantification would require in vitro or in vivo bioaccessibility studies [26].

BP offers favorable supply-side feasibility because pomace is generated at scale by juice processors; unit operations (dewatering, drying; freeze/air/spray; milling, sieving, and blending) are standard in the food industry and compatible with HACCP. Reviews of fruit-waste valorization and circular biorefineries indicate that stabilized pomace powders are among the most straightforward value-added routes, with relatively low capital intensity compared with solvent-based extracts, provided moisture is reduced sufficiently to ensure microbial stability and pigment retention [60,61]. In fermented dairy, techno-economic considerations include powder cost (linked to drying energy and yield), homogenization/viscosity impacts (which may reduce the need for added stabilizers), and consumer acceptance of color/astringency. Prior studies with apple pomace and berry pomaces report that small-to-moderate inclusion levels (≈0.5–2% for fine powders, higher for syrups/pulps) are organoleptically acceptable and can replace part of texture-modifying additives benefits that can offset ingredient costs [35,47,48]. Given the enhanced products’ positive sensory outcomes and microstructural densification with BP addition, a pilot-scale run using industrial milling and controlled-atmosphere packaging would be a realistic next step, supported by kinetic stability data to define inventory turns and minimize write-offs.

### 3.3. Physicochemical Characterization of Supplemented Stirred Yogurt Samples

A physicochemical examination was performed on the nutritional characteristics of the BP-containing stirred yogurts and the control sample. Table 4 delineates the physicochemical characteristics of the control and experimental stirred yogurts. Adding BP powder to the enriched stirred yogurts markedly improved its chemical composition compared to the control sample. A significant difference (*p* < 0.05) was observed in the proximate makeup of the stirred yogurt samples containing BP compared to those without it. The incorporation of BP causes significant variations (*p* < 0.05) in the concentrations of total dry matter, fat, carbohydrates, protein, fiber, ash, moisture, and energy value.

In addition, the enrichment of stirred yogurt with BP led to a moisture decrease, which was followed by increases in protein, fat, fiber, carbohydrates, ash, and total dry matter as the level of BP addition increased. This occurred because the incorporation of BP increased the proportion of solid components in the yogurt formulation, thereby reducing the relative water content and concentrating other nutrients such as proteins, lipids, fiber, carbohydrates, ash, and total dry matter. The significant levels of dietary fiber and crude protein in BP powder may account for these results [46,52].

The total dry matter content increased proportionally (*p* < 0.05) with the concentration of BP. The dry matter content of a product reflects its quality, nutritional value, and storage relevance. In other study the dry matter varied between 15.52 ± 0.09 and 17.76 ± 0.03 and the ash content from 0.24 ± 0.06 to 0.30 ± 0.08 for the smoothies with 3% and 6% dried BP [16].

In contrast, the fat content showed no significant variation among the enriched samples. The moisture content decreased with the increasing concentration of BP. The control sample contained 84.99% moisture, whereas DBBP3 showed the lowest moisture content at 79.54%, suggesting that the solid content of the stirred yogurt increased with the addition of BP.

The ash content, which is indicative of the mineral content, increased with the incorporation of BP, reaching 1.01% in DBBP3, compared to 0.88% in the control. This suggests that BP contributes essential minerals to the stirred yogurt.

A significant increase in protein content was observed with the addition of BP. The protein content rose from 3.45% in the control to 4.21% in DBBP3, highlighting the beneficial effect of BP as a source of protein. The fiber content was notably enhanced in the enriched samples, with DBBP1 showing 3.71%, DBBP2 at 6.99%, and DBBP3 reaching 9.97%, indicating that BP contributes significantly to the dietary fiber content of the stirred yogurt. Likewise, Italian Vastedda cheese enriched with 1% (w/w) red grape pomace powder and produced using four lactic acid bacteria strains and ovine milk exhibited a reduction in fat content, an increase in protein content, and elevated levels of secondary lipid oxidation [61].

In terms of the carbohydrates, the differences were significant (*p* < 0.05) between the enriched samples (DBBP1, DBBP2, DBBP3). Thus, a slight increase in carbohydrate content was noted across the enriched samples, with DBBP3 having the highest value (11.93%). This reflects the natural sugar content of the BP. El-Moneim [62] reported that the incorporation of 10–50% brewer’s spent grain powder into processed cheese formulations resulted in increased fiber content, total solids, pH, meltability, texture parameters, and sensory scores, while moisture, titratable acidity, protein, and ash levels decreased [62].

The results of the proximate chemical composition analysis demonstrated that using a powdered bioactive component during stirred yogurt production significantly enhanced the nutritional quality relative to the control sample.

The factors that indicate acidity, levels of total sugars, and sweetness, and therefore affect sensory features chosen for direct consumption and industrialization, include pH, titratable acidity (TTA), total soluble solids (TSSs), and the ratio of TSS/TTA [57].

The pH value is a crucial indicator for evaluating the quality of stirred yogurts, since it directly impacts the storage conditions and preservation techniques. There was a significant difference (*p* < 0.05) in the pH levels between the control and treated samples (Table 5). When BP powder was added to enriched stirred yogurts, the pH values of the formulated samples were higher than those of the control samples. The DBBP3 (15%) samples containing 15% powder had the highest pH value, while the control sample, DBC, had the lowest pH values. Therefore, BP caused a slight increase in pH to 4.99 ± 0.03 (DBBP1), 5.02 ± 0.04 (DBBP2), and 5.08 ± 0.07 (DBBP3) compared with the control (4.57 ± 0.02) (DBC), which was due to the neutral pH of BP (pH = 6.06). Nevertheless, the pH of all samples decreased after storage, with a statistically significant difference (*p* < 0.05). On day 21, the pH levels of the treatments and the control ranged from 4.32 to 4.73.

There was a statistically significant drop (*p* < 0.05) in the pH value of the formulations after storage. This decrease was caused by increased titratable acidity, which is inversely related to pH. The present investigation corroborates the findings of Chavan et al. [63]. They produced a mango-based beverage with whey as the foundation, and after 30 days of refrigeration, they saw a noticeable drop in pH from 4.3 to 4.1.

The acidity of the control stirred yogurt (0.55%) was higher than that of DBBP1 (0.50%), DBBP2 (0.49%), and DBBP3 (0.47%). BP powder did not affect the acidity of the supplemented stirred yogurts (*p* > 0.05) compared to the control. At the end of storage, BP significantly increased the acidity of the enriched stirred yogurts reaching 0.60, 0.54, 0.53, and 0.50% in the control, DBBP1, DBBP2, and DBBP3, respectively. Abdo et al. [64], reported similar trends in the pH and acidity of stirred yogurt beverages enriched with beetroot stalk extract as a functional colorant.

The BP powder influenced the TSS of the stirred yogurt, as the changes among treatments (DBBP1, DBBP2, and DBBP3) and the control sample were significant, being in a range of 7.10–8.90 on day 0, so, the TSS content gradually increased with powder addition. By the end of storage, all stirred yogurts’ TSS increased significantly. The rise in TSS might have been because insoluble polysaccharides were being changed into reduced sugars. Acid hydrolysis of sugars can also raise the amounts of reducing sugars. This may have happened because disaccharides were broken down into monosaccharides. Another possible explanation for the sudden rise in TSS levels could be the hydrolysis of sucrose into inverted sugars [65].

Alane et al. [66] discovered a similar rising trend in TSS levels in a whey-based mango herbal beverage. They found that the TSS concentration increased from 15 to 17.2 °Brix after 30 days of storage. According to the authors, a rise in TSS might arise from the insoluble portion of the product dissolving in ascorbic and citric acid while being stored. The increase in TSS was also reported in other studies on a whey-based orange beverage [67], whey-based papaya ready-to-serve beverage [68], and whey-based banana herbal (*Mentha arvensis*) beverage [69].

On day 0, the TSS/TTA ratio was in a range of 12.91 ± 0.03–18.94 ± 0.07. The TSS/TTA was increased significantly by increasing the BP powder compared to the control. The higher concentration of the powder, the lower the acceptability (Table 5). The TSS/TTA ratio of the stirred yogurts was reduced significantly on day 21. Furthermore, the ratio parameter quantifies the correlation between TSS and TTA and is employed to measure the level of sweetness of the product [70].

Scientific research using projective methodologies have found that there are multiple aspects that impact how consumers perceive and accept new food products [71,72]. Among these factors, it is worth mentioning those that are associated with the color of the product.

### 3.4. Color Evaluation of Supplemented Stirred Yogurts

The color substantially influences the consumer perception of dairy products, a factor that is as crucial as their shelf life. Fruits rich in anthocyanins are commonly used as natural alternatives to synthetic food colorants like FD&C Red 40 and carmine in products such as jellies, stirred yogurts, and confectioneries [73].

Blackcurrant pomace is a plant that is abundant in colors, including anthocyanins, and compounds that are phenolics and flavonoids [8].

Table 6 shows the color measurement data (L*, a*, b*) for stirred yogurt samples on the first control day and 21 days after storage at 4 °C.

The L* values, which reflect the lightness of the product, showed a gradual decrease with increasing levels of BP addition. Analysis of the color parameters revealed that the addition of BP significantly influenced the visual appearance of the stirred yogurts. The use of dried BP resulted in significantly darker smoothies when the concentration increased from 3% (L* = 37.50) to 6% (L* = 35.31) BP [16].

Specifically, the experimental formulations exhibited lower lightness (L) and blueness (b) values, alongside higher redness (a) values*, compared to the control. Over the 21-day storage period, statistically significant changes (*p* < 0.05) were observed in L*, a*, and b* parameters.

Chroma is the quantitative attribute of color intensity, and hue angle gives the qualitative attributes of colors which are defined as reddish, greenish, yellowish, and bluish. Chroma or intensity indicates the purity of the color. The Chroma values were highest in DBBP31 (0.47 ± 0.180), indicating more vivid coloration. Hue angle values close to 360° confirmed that the resulting color tone of both stirred yogurt samples remained in the red spectrum, as a hue angle of 0° or 360° typically corresponds to red, whereas 90°, 180°, and 270° represent yellow, green, and blue, respectively [74]. There were no significant changes in the hue angle during the storage period.

ΔE, which represents the overall color change, increased progressively with higher levels of chokeberry pomace addition and further intensified during storage. The incorporation of BP powder significantly influenced (*p* < 0.05) the ΔE values of both control and BP-enriched stirred yogurts. However, an increase in the ratio of BP from 5% to 12% dramatically elevated the ΔE. The color change is most prominent in DBBP3, with the addition of BP contributing to an increasingly intense red color over time.

The addition of BP powder resulted in a progressively darker color in the supplemented stirred yogurts as the ratio of BP powder increased (*p* < 0.05). As the proportion of BP powder grew, the color of the stirred yogurts moved closer to zero, while the control stirred yogurt, which had the lightest color, exhibited the highest L* value. Smoothies enriched with BP showed a noticeably deeper red color, with each added dose significantly increasing color intensity. Over time, storage led to a marked decrease in the a* value. These color changes, affected by both pomace concentration and storage, mainly reflect the instability of anthocyanins [75].

The a* values of the stirred yogurt samples changed significantly (*p* < 0.05) when BP powder was added. DBBP3 containing 15% BP powder appeared to be closer to the color red than the control sample, which was perceived as being closer to the color green. The predominant hue of stirred yogurt containing blackcurrant was red, which was caused by the conversion of anthocyanins at low pH values. The a* values across all BP-enriched samples show a significant increase in redness over time, with the intensity of red being more prominent in DBBP3, followed by DBBP2 and DBBP1.

It was found that the control stirred yogurt’s b* values were fairly near to the hue yellow. The hue of the stirred yogurt significantly approximated blue after the addition of BP powder (*p* < 0.05). For all BP-enriched samples, the b values* increase, over the storage period. The control sample (DBC) shows the highest increase in yellowness.

Yogurt beverages enriched with salal berries pomace have a positive a* value, indicating red coloration, while both control and BP samples display a negative axis coordinate. The control stirred yogurt exhibits the greatest L* value, indicating that it possesses the brightest color, succeeded by BP and salal berries pomace. The overall anthocyanin concentration diminished progressively with prolonged storage duration, consistent with earlier studies [15].

### 3.5. Mineral Profile

The mineral profile, particularly macroelements, plays a crucial role in determining the nutritional value and health benefits of any enriched food. In the case of a dairy stirred yogurt enriched with BP, the presence and balance of key macroelements such as calcium, phosphorus, magnesium, sodium, and potassium are essential for providing both functional and health-promoting properties [76].

Table 7 presents the mineral content analysis for the control stirred yogurt sample without BP and the BP-supplemented stirred yogurt samples at three distinct percentages (5%, 10%, and 15%).

The amounts of mineral content increased in congruence with the increased amount of BP powder. The differences between the stirred yogurt samples in terms of the amounts of Na, Mg, P, K, Ca, Fe, Cu, and Zn were found to be statistically significant (*p* < 0.05). Stirred yogurt samples contained additional essential elements, including calcium and phosphorus. Notably, the calcium content showed a progressive increase from 117.67 mg/100 g to 137.22 mg/100 g in the BP-supplemented stirred yogurts. A similar trend is observed with phosphorus, where the concentration increases as the proportion of BP in the stirred yogurt rises. The values range from 27.67 mg/100 g in DBC (control) to 73.51 mg/100 g in DBBP3, indicating that the addition of BP positively influences the phosphorus levels.

Potassium content shows a notable increase as well, with values escalating from 168.63 mg/100 g in DBC to 270.12 mg/100 g in DBBP3.

The magnesium levels followed a similar trend, with higher values observed in the DBBP2 and DBBP3 samples. The magnesium concentration increased from 55.28 mg/100 g in the control sample to 76.23 mg/100 g in the 15% BP-supplemented stirred yogurt. Sodium content increased significantly with higher concentrations of blackcurrant pomace, from 110.30 mg/100 g in DBC to 144.22 mg/100 g in DBBP3.

Zinc content also increased as the BP percentage rose, though the values for DBBP3 (0.70 mg/100 g) were not significantly different from DBBP2 (0.64 mg/100 g). The increase in zinc content demonstrates that BP can be a valuable source of this essential micronutrient. A noticeable increase in iron content was observed in the BP-supplemented samples, with values ranging from 4.41 mg/100 g in DBC to 7.81 mg/100 g in DBBP3. Copper levels also showed an increase with the addition of blackcurrant pomace, from 0.09 mg/100 g in DBC to 0.15 mg/100 g in DBBP3.

The study’s findings showed that adding powdered BP raw material to stirred yogurt increased the quantity of minerals in the final products. As can be seen in Table 2, freeze-dried BP powder is rich in Ca, K, Mg, P, and Na. Hence, BP powder is suitable for incorporation as a bioactive ingredient in foods.

Additionally, it was observed that adding concentrated freeze-dried black carrot fiber at different ratios (0%, 0.25%, 0.5%, and 1%) to an ayran-containing black carrot fiber enhanced its Ca, K, Mg, Na, P, and Zn levels significantly [77].

Goosen et al. [78] found that the fruit juice of the indigenous African sour plum had similar levels of K (525 mg/100 g), lower levels of Ca (3.04 mg/100 g), Mg (15.9 mg/100 g), P (24.6 mg/100 g), Na (0.82 mg/100 g), and higher levels of Fe (170 mg/100 g).

### 3.6. Microstructure Analysis

**Figure 1:**
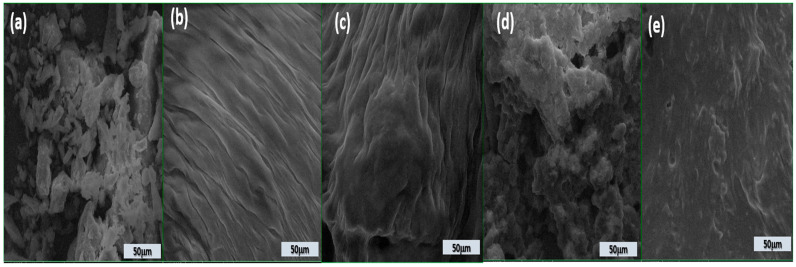
Scanning electron microscopy micrographs of stirred yogurt enriched with BP powder (**a**); DBC stirred yogurt without powder addition (**b**); DBBP1 (**c**); DBBP2 (**d**); and DBBP3 (**e**) stirred yogurt with 5, 10, and 15% powder of BP, respectively.

The microstructure analysis (Figure 1) of dairy stirred yogurts enriched with BP, as observed through scanning electron microscopy (SEM) at a scale of 50 µm, shows notable differences based on the level of BP incorporation.

The stirred yogurts containing BP powder (Figure 1c–e) exhibit a unique microstructure compared to the control stirred yogurt (Figure 1b).

The structure of BP powder (Figure 1a) appears fragmented and irregular with loosely arranged particles, indicating a weak and unstructured matrix. Several gaps and pores are visible; the control’s surface is uneven. This disordered appearance is typical of stirred yogurts lacking stabilizing or fiber-rich components like pomace powder.

The addition of 5% BP (Figure 1c) results in a more organized, layered structure. The presence of fine, aligned fibrous layers suggests partial integration of the pomace fiber into the protein matrix, improving structural coherence. The addition of BP significantly diminished the pore gaps and channels in the stirred yogurt samples, resulting in denser and more homogenous protein aggregation and microstructure. BP particles may exist in the voids around the casein aggregates.

At 10% BP, the matrix becomes more compact and denser. The fibrous texture is intensified, reflecting better incorporation and distribution of the BP within the stirred yogurt, leading to improved network formation and potential enhancement in textural quality.

A rough and heterogeneous surface is observed at 15% BP addition. The dense accumulation of particles shows strong interactions but may also point to reduced homogeneity due to excess fiber, which could affect smoothness and sensory perception.

Figure 1e confirms that while the structure remains dense, it appears smoother than Figure 1d, potentially due to the collapse of the microstructure or tight packing of fibers and proteins at high BP levels.

The incorporation of BP into the stirred yogurt produced a more homogeneous and uniformly distributed casein network, resulting in a smoother texture and reduced gaps. Nonetheless, the general texture of the stirred yogurt seemed somewhat coarser. This occurrence can be ascribed to the association between BP and milk proteins, enabled by hydrocolloids and the emulsion’s stability.

The findings align with the discoveries of Ibrahim and Khalifa [79]. The dietary fiber of BP can bind water and enhance the water-holding capacity of stirred yogurt samples, hence improving protein linkages [80].

**Figure 2:**
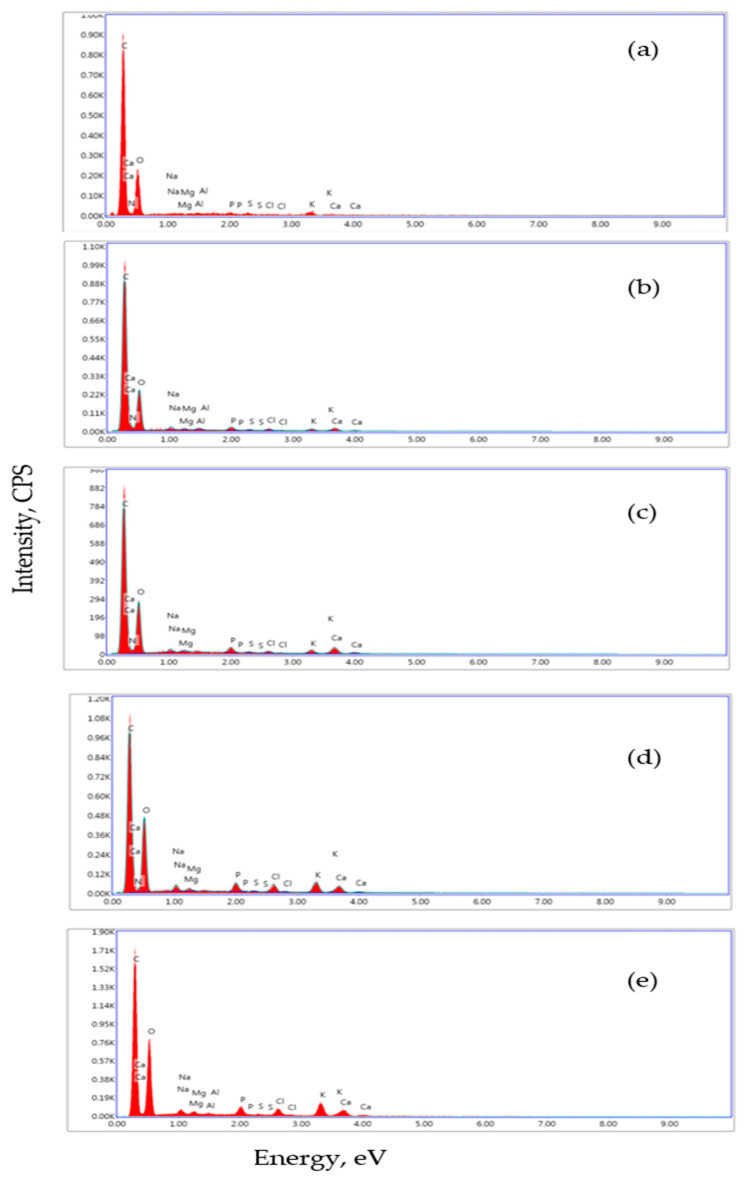
EDX analysis of BP (**a**), DBC (**b**), DBBP1 (**c**), DBBP2 (**d**), and DBBP3 (**e**).

Figure 2a presents the EDX spectrum of BP, indicating the presence of alkali elements (potassium, sodium) and alkaline earth ions (magnesium) on the surface, consistent with Table 2. The EDX spectrum of the supplemented stirred yogurts indicates the presence of calcium, magnesium, and phosphorus (Figure 2b–e).

### 3.7. Microbial Analysis of Supplemented Stirred Yogurts

Table 8 presents a detailed summary of the microbiological parameters assessed in both control (non-enriched) and BP-enriched stirred yogurt samples, in which BP was incorporated at three concentrations: 5%, 10%, and 15% (denoted DBBP1, DBBP2, and DBBP3, respectively). The microbiological analysis was conducted to evaluate the microbial stability and safety of these stirred yogurts over a 21-day storage period at 4 °C.

The results clearly demonstrate that all stirred yogurt formulations remained microbiologically safe for consumption throughout the entire storage duration. Notably, neither *Escherichia coli* nor coagulase-positive *Staphylococci* were detected in any sample, at either the initial day (day 0) or after 21 days of cold storage, indicating the effectiveness of the thermal treatment and hygienic production conditions.

Over the 21-day period, a gradual increase in microbial counts was observed across all formulations, encompassing total aerobic bacteria (TAB), yeasts, molds, and coliforms. This trend is consistent with the natural progression of microbial growth in minimally preserved stirred yogurts, particularly those stored under refrigeration. Nonetheless, the growth remained within acceptable safety thresholds, with the highest recorded values being 8.97 log CFU/mL for TAB, 1.78 log CFU/mL for coliforms and 2.27 log CFU/mL for molds in DBBP3 after 21 days. In another study, microbiological analysis confirmed the absence of *Enterobacteriaceae*, yeasts, and molds in the buttermilk supplemented with 2% and 10% blueberry pomace [28].

A gradual increase in microbial counts was observed across all formulations, encompassing total aerobic bacteria (TAB), yeasts, molds, and coliforms, with the highest counts recorded in DBBP3 (8.97 log CFU/mL TAB; 1.78 log CFU/mL coliforms; 2.27 log CFU/mL molds at day 21). This trend is consistent with the natural progression of microbial growth in minimally preserved fermented dairy products [47]. The increase in yeast, mold, and coliform counts with higher BP concentrations may be attributed to the natural epiphytic microflora associated with blackcurrant pomace, which can harbor native yeasts and environmental bacteria originating from the surface of the berry [81]. Although microbial proliferation was more evident in DBBP3, levels remained within the microbiological safety limits established for fermented dairy products stored under refrigeration.

Additionally, BP contains phenolic compounds with mild antimicrobial properties, which may modulate microbial growth and delay spoilage [82]. To further enhance shelf-life stability, mitigation strategies may include UV-C or mild heat treatment of BP prior to incorporation, the use of protective or bioprotective cultures, and rigorous hygienic handling [83]. These findings support the feasibility of BP incorporation at levels up to 15% without compromising microbiological safety, while preserving the product’s functional potential.

It is also worth noting that the presence of BP powder may exert a mild antimicrobial effect, potentially influencing the dynamics of microbial proliferation in enriched variants [59]. The incorporation of BP at up to 15% does not compromise the microbiological safety of the supplemented stirred yogurt. However, this antimicrobial activity, which is largely attributable to phenolic compounds in the pomace, could interact with fermentation microorganisms and thereby modulate the structure and texture by altering microbial growth or metabolic activity. Studies have demonstrated that dietary polyphenols possess both antimicrobial and selective modulation effects on microbes, suppressing undesirable bacteria while promoting beneficial ones [84].

When stored at 4 °C, the products maintain acceptable microbial quality for a minimum of 21 days, thereby supporting the viability of BP-enriched stirred yogurts in commercial functional food applications.

### 3.8. Sensorial Analysis of Supplemented Stirred Yogurts

**Figure 3:**
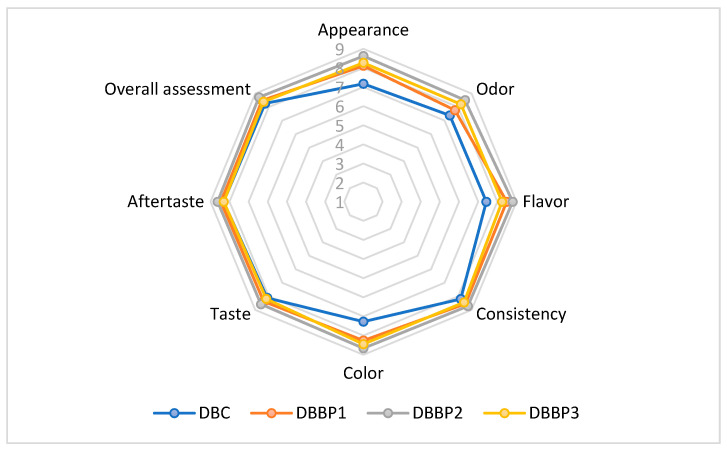
Comparative diagram of the sensory attributes specific to supplemented stirred yogurts (DBC stirred yogurt without powder addition); DBBP1; DBBP2; and DBBP3 stirred yogurt with 5, 10, and 15% powder of BP).

The sensory evaluation of the supplemented stirred yogurts was conducted using a nine-point hedonic scale. The powder was added to stirred yogurt at 5%, 10%, and 15% (DBBP1–DBBP3), with a control sample (DBC) containing no BP powder. The average scores from the sensory assessment are shown in Figure 3. The evaluated attributes included appearance, color, flavor, taste, texture, odor, aftertaste, and overall acceptability.

The enriched stirred yogurts that contained 5%, 10%, and 15% BP powder showed higher ratings for sensory characteristics in comparison to the control.

**Figure 4:**
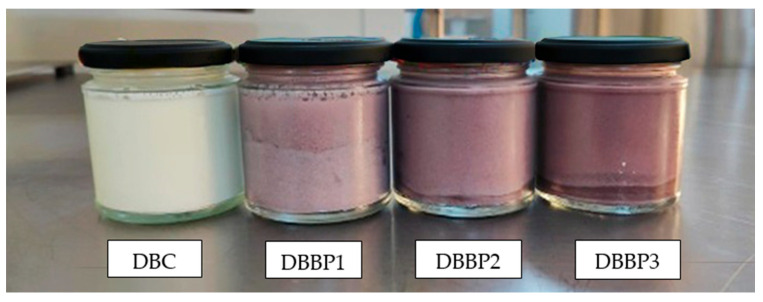
Dairy stirred yogurt samples with different percentages of BP powder: control (DBC), 5% (DBBP1), 10% (DBBP2) and 15% (DBBP3).

The aesthetic quality of the stirred yogurts fluctuated with the degree of BP addition. The control sample (DBC) retained a characteristic milky look, whereas the BP-enriched samples displayed increasingly intense purple shades attributed to the anthocyanins in the BP (Figure 4). DBBP1 exhibited a subtle purplish hue, which was predominantly seen as attractive. DBBP2 had a vivid, more intense hue, receiving the maximum rating in this category. Nonetheless, DBBP3, with 15% BP, had a too dark and rather opaque quality, which many panelists considered less visually appealing.

The perception of flavor is influenced by a combination of sensory factors, including taste, aroma, and texture. DBC displayed a neutral, creamy dairy taste. DBBP1 exhibited a subtle fruity boost that matched the dairy foundation. DBBP2 attained the highest ratings for flavor, harmonizing the acidity of blackcurrant with the richness of dairy. DBBP3, however, exhibited a dominant sour and mildly bitter flavor that masked the inherent dairy taste, resulting in diminished acceptability in this category. All samples maintained a smooth, palatable consistency; however, the use of BP slightly modified the mouthfeel. DBBP1 and DBBP2 were characterized as smooth with a subtle pulpy texture that augmented sensory complexity. DBBP3, owing to its elevated fiber content, was regarded as excessively thick or gritty by certain panelists, diminishing its textural appeal.

Odor assessment indicated that DBC possessed a typical dairy aroma devoid of any off notes. The addition of BP imparted a delightful, fruity fragrance to DBBP1 and DBBP2, enhancing their sensory appeal. Conversely, DBBP3’s high pomace concentration generated a more pronounced and earthy aroma that was less favored. The impression of aftertaste exhibited the same pattern. DBBP1 and DBBP2 were characterized by a revitalizing, mildly acidic aftertaste. Nonetheless, DBBP3 imparted a more astringent and slightly bitter aftertaste which influenced the overall experience.

Among all samples, DBBP2 (10% BP) attained the highest ratings for overall acceptability. It offered a harmonious sensory profile characterized by attractive color, intensified flavor, and agreeable aroma, without overpowering the foundational stirred yogurt. DBBP1 (5% BP) garnered positive feedback, particularly from individuals favoring a more understated fruit profile. DBBP3 (15% BP) received the lowest grade mostly because of its overpowering taste, odor, and viscous texture, which diminished the stirred yogurt’s appeal.

**Figure 5:**
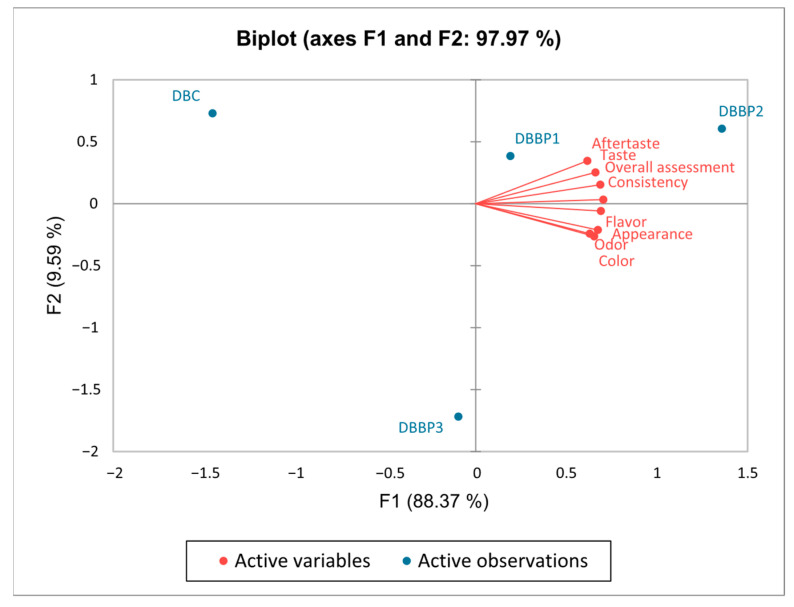
Representation of correlations between sensory attributes using principal component analysis (PCA).

The PCA biplot in Figure 5 visually illustrates the positioning of the four stirred yogurt samples (plain and those flavored with BP) based on the sensory attributes outlined in Figure 4. The first principal component (PC1) explained 88.37% of the variation and primarily represented the three dairy stirred yogurt samples containing BP. The second component represented 9.59% of the total variance and was composed solely of control stirred yogurt. Together, the two axes explained 97.97% of the overall variation. On the first axis (F1), the attributes of taste, aftertaste, consistency, and overall assessment were strongly correlated. Likewise, the attributes of appearance, color, flavor and odor also showed a positive correlation in the same quadrant of the first axis (F1). Since all sensory qualities were associated only with the same axis (F1), control stirred yogurt was regarded as neutral but the DBBP3 sample was considered less positive. The biplot analysis facilitates precise localization of the four stirred yogurt samples based on their sensory attributes, allowing for differentiation through positive correlations between sensory qualities and the dairy stirred yogurt samples.

According to Figure 4, the stirred yogurt samples with a 5% and 10% BP content proved more satisfactory compared to samples with 15% BP. All sensory properties achieved the highest degree of acceptance at these percentages. All of the proposed samples were positively evaluated by the panelists, with DBBP2 being favored.

The optimal sensory experiences were achieved with a 10% incorporation of defatted blackcurrant seeds, but a 30% addition markedly diminished overall consumer approval [85]. A study by Blejan et al. [86] developed new fruit leathers by incorporating 0.5%, 1.0%, and 1.5% BP powders into pear leather formulations, which also included honey (5%), pectin (1%), and lemon juice (2.5%) as additional ingredients. These newly developed fruit leathers can be recommended as alternative snack foods with high nutritional value and functional properties.

All samples of stirred yogurt enriched with BP powder demonstrated positive sensory assessments. Panelists determined that the incorporation of 10% BP into the stirred yogurt yielded the most favorable enhancement, surpassing the outcomes of other treated stirred yogurt products.

## 4. Conclusions

The findings of this study indicate that blackcurrant pomace (BP) powder is a valuable source of biologically active compounds, particularly anthocyanins and fibers, which exhibit substantial antioxidant activity. The incorporation of BP powder into stirred yogurt significantly enhanced the bioactive compound contents and antioxidant capacity, thereby improving its overall nutritional profile particularly by increasing its fiber and carbohydrate content. The incorporation of up to 15% blackcurrant pomace in stirred yogurt maintained microbiological safety during 21 days of refrigerated storage, with no detection of pathogenic bacteria and only moderate increases in spoilage microorganisms, likely moderated by the mild antimicrobial effect of pomace phenolics. Compared with the control formulation, BP-enriched yogurt demonstrated a marked increase in mineral content (Mg, P, K, Ca, and Na), attributable to the contribution of the dry bioactive ingredient. Sensory analysis showed that panelists appreciated the enhanced color intensity, resulting in a higher overall acceptance of the enhanced samples. The stirred yogurt containing 10% BP powder (DBBP2) displayed the most favorable composition and sensory characteristics while demonstrating good potential for consumer acceptance as a functional, sustainable dairy product.

The abundant nutritional composition of dried BP powder (anthocyanins, polyphenols, and minerals) provides opportunities to develop aesthetically appealing, functional dairy products with potential health benefits. Moreover, the use of BP derived from juice processing by-products supports the circular economy and offers a sustainable alternative to synthetic colorants and antioxidants in dairy formulations.

Regarding the limitations and future perspectives, this study was conducted at a laboratory scale, with a storage period limited to 21 days and without in vivo or consumer validation. The bioavailability and stability of anthocyanins and other bioactives were not evaluated beyond analytical quantification. Future research should focus on scaling up the process to an industrial level, extending the shelf-life assessment beyond 21 days, and conducting in vitro or in vivo studies to evaluate bioaccessibility, consumer acceptance, and techno-economic feasibility.

## Figures and Tables

**Table 1:** Characterization of the experimental batches.

Batches	Description
DBC	Stirred yogurt—product without added ingredients
DBBP1	Stirred yogurt with 5% BP powder
DBBP2	Stirred yogurt with 10% BP powder
DBBP3	Stirred yogurt with 15% BP powder

**Table 2:** Phytochemical and physicochemical profile of BP powder.

Parameter	BP Powder
Total Anthocyanins, mg C3G/g DM	3.14 ± 0.09
Total Flavonoids, mg CE/g DM	5.16 ± 0.10
Total Polyphenols, mg GAE/g DM	9.83 ± 0.12
Antioxidant activity, µmol TE/g DM	24.52 ± 0.83
Inhibition, %	92.48 ± 1.23
L*	34.73 ± 0.18
a*	19.48 ± 0.13
b*	6.95 ± 0.07
Chroma	20.68 ± 0.15
Hue angle	0.34 ± 0.01
Moisture, %	10.28 ± 0.15
Ash, %	1.04 ± 0.11
Lipids, %	4.18 ± 0.13
Proteins, %	4.68 ± 0.24
Total dietary fiber, %	15.48 ± 0.44
Calcium (Ca, mg/100 g)	289.65 ± 0.31
Phosphorus (P, mg/100 g)	49.14 ± 0.24
Potassium (K, mg/100 g)	367.45 ± 0.34
Magnesium (Mg, mg/100 g)	101.22 ± 0.27
Zinc (Zn, mg/100 g)	1.39 ± 0.20
Iron (Fe, mg/100 g)	52.67 ± 0.29
Copper (Cu, mg/100 g)	0.94 ± 0.15
Sodium (Na, mg/100 g)	9.65 ± 0.30

L* (lightness /darkness), a* (red/greenness), and b* (yellow/blueness).

**Table 3:** Phytochemical characteristics and antioxidant activity of control and supplemented stirred yogurt and storage stability.

Phytochemical Characteristics	Storage Time, (days)	DBC	DBBP1	DBBP2	DBBP3
Total Anthocyanins, mg C3G/100 g DM	0	-	33.42 ± 0.26 ^aC^	64.52 ± 0.32 ^aB^	98.44 ± 0.41 ^aA^
	21	-	29.39 ± 0.20 ^bC^	60.41 ± 0.28 ^bB^	94.21 ± 0.33 ^bA^
Total Flavonoids mg CE/100 g DM	0	54.92 ± 1.41 ^aD^	77.61 ± 1.15 ^aC^	89.55 ± 1.57 ^aB^	106.96 ± 1.84 ^aA^
	21	51.04 ± 1.10 ^bD^	73.41 ± 1.06 ^bC^	85.49 ± 1.19 ^bB^	102.82 ± 1.28 ^bA^
Total Polyphenols mg GAE/100 g DM	0	88.53 ± 1.57 ^aD^	134.49 ± 1.68 ^aC^	154.19 ± 1.77 ^aB^	171.45 ± 1.79 ^aA^
	21	84.61 ± 1.06 ^bD^	130.55 ± 1.17 ^bC^	150.24 ± 1.19 ^bB^	167.54 ± 1.22 ^bA^
Antioxidant activity, µmol TE/g DM	0	8.21 ± 0.35 ^aD^	12.89 ± 0.41 ^aC^	16.55 ± 0.46 ^aB^	21.15 ± 0.49 ^aA^
	21	5.11 ± 0.20 ^bD^	9.78 ± 0.21 ^bC^	13.42 ± 0.25 ^bB^	18.07 ± 0.27 ^bA^

Mean values in the same column that are accompanied by various lowercase superscript letter (a, b) are substantially different (*p* < 0.05). Mean values with distinct uppercase superscript letter (A–D) in the same row are substantially different (*p* < 0.05).

**Table 4:** Physicochemical characteristics of control and supplemented stirred yogurts.

Physical–Chemical Characteristics	DBC	DBBP1	DBBP2	DBBP3
Total dry matter, %	15.01 ± 0.18 ^d^	15.89 ± 0.20 ^c^	17.84 ± 0.23 ^b^	20.46 ± 0.25 ^a^
Fat, %	3.03 ± 0.11 ^a^	3.19 ± 0.08 ^a^	3.26 ± 0.06 ^a^	3.31 ± 0.05 ^a^
Protein, %	3.45 ± 0.17 ^d^	3.78 ± 0.04 ^c^	3.98 ± 0.03 ^b^	4.21 ± 0.12 ^a^
Fiber, %	0.00 ± 0.00 ^d^	3.71 ± 0.09 ^c^	5.65 ± 0.11 ^b^	7.97 ± 0.15 ^a^
Carbohydrates, %	7.65 ± 0.12 ^c^	8.01 ± 0.09 ^c^	9.64 ± 0.15 ^b^	11.93 ± 0.16 ^a^
Moisture, %	84.99 ± 0.27 ^a^	84.11 ± 0.21 ^b^	82.16 ± 0.23 ^c^	79.54 ± 0.25 ^d^
Ash, %	0.88 ± 0.02 ^c^	0.92 ± 0.02 ^b^	0.96 ± 0.03 ^b^	1.01 ± 0.03 ^a^
Energetic value, Kcal/100 g	64.77 ± 0.09 ^d^	83.29 ± 0.11 ^c^	95.12 ± 0.14 ^b^	110.29 ± 0.16 ^a^
KJ/100 g	265.56 ± 0.09 ^d^	341.49 ± 0.11 ^c^	389.99 ± 0.14 ^b^	452.19 ± 0.16 ^a^

For each physicochemical parameter and sample, values that do not share the same lowercase superscript letter (a–d) are significantly different at *p* < 0.05.

**Table 5:** Physical–chemical analysis of control and enriched stirred yogurts at time zero and after 21 days of storage.

Physical–Chemical Characteristics	Storage Time (Day)	DBC	DBBP1	DBBP2	DBBP3
pH	0	4.57 ± 0.02 ^aC^	4.99 ± 0.03 ^aB^	5.02 ± 0.04 ^aB^	5.08 ± 0.07 ^aA^
	21	4.32 ± 0.06 ^bC^	4.55 ± 0.02 ^bB^	4.60 ± 0.03 ^bB^	4.73 ± 0.04 ^bA^
Total titratable acidity (TTA), %	0	0.55 ± 0.05 ^aA^	0.50 ± 0.08 ^aB^	0.49 ± 0.04 ^aB^	0.47 ± 0.02 ^aB^
	21	0.60 ± 0.04 ^bA^	0.54 ± 0.02 ^bB^	0.53 ± 0.06 ^bB^	0.50 ± 0.05 ^bB^
Total soluble solids (TSSs), °Brix	0	7.10 ± 0.02 ^aC^	7.50 ± 0.04 ^aC^	7.90 ± 0.03 ^aB^	8.90 ± 0.07 ^aA^
	21	7.47 ± 0.03 ^bC^	7.85 ± 0.03 ^bC^	8.32 ± 0.05 ^bB^	9.40 ± 0.06 ^bA^
TSS/TTA ratio	0	12.91 ± 0.03 ^aD^	15.00 ± 0.04 ^aC^	16.12 ± 0.06 ^aB^	18.94 ± 0.07 ^aA^
	21	12.45 ± 0.02 ^bD^	14.54 ± 0.03 ^bC^	15.70 ± 0.05 ^bB^	18.80 ± 0.06 ^bA^

Mean values with distinct superscript lowercase letter (a, b) in the same column exhibit significant differences (*p* < 0.05). The mean values with different superscript uppercase letter (A–D) in the same row are statistically distinct (*p* < 0.05).

**Table 6:** Colorimetric attributes of control and stirred yogurts enriched with BP powder during storage.

Samples	Storage Time (day)	L*	a*	b*	Chroma	Hue Angle	ΔE
DBC	0	96.12 ± 0.28 ^aA^	−3.92 ± 0.05 ^aD^	10.84 ± 0.20 ^aA^	11.52 ± 0.21 ^aA^	178.77 ± 0.04 ^aB^	-
	21	95.67 ± 0.25 ^bA^	−3.13 ± 0.04 ^bD^	11.89 ± 0.22 ^bA^	12.29 ± 0.23 ^bA^	178.68 ± 0.03 ^aB^	-
DBBP1	0	51.95 ± 0.22 ^aB^	6.73 ± 0.19 ^aC^	−0.16 ± 0.04 ^aD^	6.73 ± 0.17 ^aD^	359.97 ± 0.03 ^aA^	46.75 ± 0.23 ^aC^
	21	49.77 ± 0.14 ^bB^	8.59 ± 0.19 ^bC^	−0.07 ± 0.02 ^bD^	8.60 ± 0.16 ^bC^	359.99 ± 0.02 ^aA^	48.86 ± 0.11 ^bC^
DBBP2	0	47.60 ± 0.23 ^aC^	9.27 ± 0.14 ^aB^	−0.71 ± 0.07 ^aC^	9.30 ± 0.15 ^aC^	359.92 ± 0.02 ^aA^	51.59 ± 0.21 ^aB^
	21	45.51 ± 0.21 ^bC^	11.22 ± 0.18 ^bB^	−0.56 ± 0.05 ^bC^	11.23 ± 0.13 ^bB^	359.95 ± 0.03 ^aA^	53.64 ± 0.20 ^aB^
DBBP3	0	44.11 ± 0.20 ^aD^	10.41 ± 0.17 ^aA^	−1.12 ± 0.09 ^aB^	10.47 ± 0.18 ^aB^	359.89 ± 0.02 ^aA^	55.26 ± 0.21 ^aA^
	21	42.05 ± 0.19 ^bD^	12.56 ± 0.20 ^bA^	−0.89 ± 0.06 ^bB^	12.23 ± 0.13 ^bA^	359.92 ± 0.02 ^aA^	57.31 ± 0.19 ^aA^

The variation in the color parameter over time is indicated by lowercase superscript letters (a, b). The color variations among the samples are emphasized using uppercase superscript letters (A–D). Values that share a lowercase or uppercase letter are not statistically different (*p* > 0.05).

**Table 7:** Mineral composition of control and supplemented stirred yogurt samples.

Parameter	DBC	DBBP1	DBBP2	DBBP3
Calcium (Ca, mg/100 g)	117.67 ± 0.31 ^c^	128.77 ± 0.34 ^b^	131.55 ± 0.35 ^a^	137.22 ± 0.37 ^a^
Phosphorus (P, mg/100 g)	27.67 ± 0.24 ^d^	39.86 ± 0.29 ^c^	59.66 ± 0.30 ^b^	73.51 ± 0.33 ^a^
Potassium (K, mg/100 g)	168.63 ± 0.36 ^d^	203.31 ± 0.30 ^c^	239.38 ± 0.33 ^b^	270.12 ± 0.39 ^a^
Magnesium (Mg, mg/100 g)	55.28 ± 0.22 ^d^	62.46 ± 0.28 ^c^	69.63 ± 0.30 ^b^	76.23 ± 0.32 ^a^
Zinc (Zn, mg/100 g)	0.49 ± 0.05 ^b^	0.57 ± 0.06 ^a^	0.64 ± 0.08 ^a^	0.70 ± 0.11 ^a^
Iron (Fe, mg/100 g)	4.41 ± 0.00 ^d^	5.29 ± 0.09 ^c^	6.35 ± 0.12 ^b^	7.81 ± 0.14 ^a^
Copper (Cu, mg/100 g)	0.09 ± 0.02 ^b^	0.11 ± 0.06 ^a^	0.13 ± 0.07 ^a^	0.15 ± 0.09 ^a^
Sodium (Na, mg/100 g)	110.30 ± 0.28 ^d^	128.72 ± 0.30 ^c^	136.41 ± 0.32 ^b^	144.22 ± 0.35 ^a^

Superscripts with distinct letters (a–d) in the same row indicate a significant difference (*p* < 0.05).

**Table 8:** Microbial quality of the control and supplemented stirred yogurt samples.

Parameter	Storage Time (Day)	DBC	DBBP1	DBBP2	DBBP3
TAB, log CFU/mL	0	6.96 ± 0.32 ^aC^	7.25 ± 0.23 ^aB^	8.23 ± 0.18 ^aA^	8.73 ± 0.19 ^aA^
	21	7.39 ± 0.19 ^bB^	7.84 ± 0.42 ^bB^	8.56 ± 0.17 ^bA^	8.97 ± 0.17 ^aA^
Yeast, log CFU/mL	0	0.86 ± 0.17 ^aD^	1.12 ± 0.13 ^aC^	1.36 ± 0.36 ^aB^	1.58 ± 0.22 ^aA^
	21	0.93 ± 0.15 ^aD^	1.28 ± 0.16 ^aC^	1.43 ± 0.21 ^aB^	1.73 ± 0.12 ^aA^
Molds, log CFU/mL	0	0.23 ± 0.16 ^aD^	1.18 ± 0.15 ^aC^	1.89 ± 0.20 ^aB^	2.15 ± 0.13 ^aA^
	21	0.26 ± 0.09 ^aD^	1.30 ± 0.21 ^aC^	2.13 ± 0.19 ^aB^	2.27 ± 0.18 ^aA^
Coliforms, log CFU/mL	0	1.47 ± 0.23 ^aC^	1.63 ± 0.18 ^aB^	1.72 ± 0.25 ^aA^	1.77 ± 0.25 ^aA^
	21	1.52 ± 0.15 ^aC^	1.67 ± 0.13 ^aB^	1.75 ± 0.05 ^aA^	1.78 ± 0.13 ^aA^
*Escherichia coli*	0	Not detected	Not detected	Not detected	Not detected
	21	Not detected	Not detected	Not detected	Not detected
Coagulase-positive staphylococci	0	Not detected	Not detected	Not detected	Not detected
	21	Not detected	Not detected	Not detected	Not detected

TAB—total aerobic bacteria count. Mean values with distinct lowercase superscript letters (a, b) in the same column exhibit significant differences (*p* < 0.05). The mean values with different uppercase superscript letters (A–D) in the same row are statistically distinct (*p* < 0.05).

## Data Availability

The original contributions presented in the study are included in the article, further inquiries can be directed to the corresponding authors.
